# The complete mitochondrial genome of *Poropanchax normani* and phylogenetic studies of Cyprinodontidaes

**DOI:** 10.1080/23802359.2021.1970633

**Published:** 2021-08-31

**Authors:** Chuanchen Ren, Xinyu Zhao, Fang Meng, Yifan Liu, Hongqiang Wang, Kun Zhang, Bingjian Liu, Enshang Yang

**Affiliations:** a National Engineering Research Center for Marine Aquaculture, Zhejiang Ocean University, Zhoushan, China; b National Engineering Laboratory of Marine Germplasm Resources Exploration and Utilization, Marine Science and Technology College, Zhejiang Ocean University, Zhoushan, China; c Zhoushan Hospital of Zhejiang Province, Zhoushan, China; d School of Marine Engineering Equipment, Zhejiang Ocean University, Zhoushan, China

**Keywords:** *Poropanchax normani*, mitochondrial genome, evolutionary relationships

## Abstract

The complete mitochondrial genome of the Cyprinodontiformes fish *Poropanchax normani* was studied in this study. The size of the entire mitochondrial genome was 16,878 bp, and the total length of the encoding sequence was 11,435 bp, accounting for 67.75%, encoding 3800 amino acids in total. Genome base compositions were: A was 27.30%, C was 28.30%, G was 16.17%, and T was 28.23%. ND2 and CO1 started with GTG, and other protein-coding genes started with ATG. ND1, ND2, and ND3 ended with TAG, respectively, CO2 and ND4 ended with a single T, and other PCGS ended with TAA. The ribosomal RNA lengths of 12S and 16S were 950 bp and 1690 bp, respectively. The control area (D-loop) was 1194 bp in size and ranged from 15,685 to 16,878 bp. It showed negative GC skew value (–0.2728) and negative AT skewness (–0.0168). Phylogenetic analysis showed that *P. normani* was most closely related to *Xenotoca eiseni*. The complete sequence of the mitochondrial genome will provide a new perspective for classification and help to draw a more complete picture of species diversity within the Cyprinodontiformes.

*Poropanchax normani* belongs to the family Cyprinodontidae and the order Cyprinodontiformes, native to the waters of a plateau lake in West Africa. *P. normani* is a small fish with a body length of about 4 cm and a weight of about 0.6 g. The adult fish has a spindle-shaped body and bright blue eyes. Mitochondrial DNA (mtDNA) is maternal inheritance and has the advantages of relatively conservative gene and relatively small sequence. Therefore, mtDNA has been widely used in evolutionary and phylogenetic studies (Zhou et al. 2019). In the present study, we determined the complete sequence of the mitochondrial genome of *P. normani*, to supply more data at the molecular level and provide a reference for further systematic research.

The specimens of *P. normani* fish were collected from plateau lakes in West Africa (6°13′7″N, 102°14′7″E) and then stored in the −80 °C refrigerator at the Engineering Research Center for Mariculture and Fishery Enhancement Museum in Zhejiang Province (accession number: PR232850). Total genomic DNA was extracted from the muscles of three different individuals using the phenol–chloroform method (Barnett and Larson 2012). COI is a protein-coding gene (PCG) on mtDNA, and COI fragments of mitochondrial genes are made into ‘barcodes’ for species identification (Hebert et al. 2003). Due to the characteristics of suitable length and slow evolution rate (Liu et al. 2010), and the COI gene of most animals can be amplified by universal primers (Table S1), we first sequenced the COI, then determined this species by blasting in NCBI. The calculation of base composition and phylo-genetic construction were conducted by MEGA6.0 software (Tamura et al. 2013). Transfer RNA (tRNA) genes were generated by the TRNAS-can-SE program (Lowe and Eddy 1997). The mitochondrial genome sequence of *P. normani* was sequenced by Sanger dideoxy with the annotated genes was stored in GenBank with the login name MW354542.

The mitogenome of *P. normani* was a closed double-stranded circular molecule of 16,878 bp including 13 PCGs, two rRNA genes, 22 tRNA genes, and two main noncoding regions, which was similar to the typical mitogenome of vertebrates (Boore 1999). The contents of A, C, G, and T were 27.30%, 28.30%, 16.17%, and 28.23%, respectively. Most mitochondrial genes were encoded on H-strand except for ND6 and eight tRNA genes (Gln, Ala, Asn, Cys, Tyr, Ser, Glu, and Pro), which were encoded on the L-strand. The proportion of coding sequences with a total length of 11,435 bp was 67.75%, 13 PCGs encoded 3800 amino acids. A-T and G-C contents of mitochondrial genome were 55.52% and 44.47%, respectively, thereby AT bias was higher. Besides, it showed negative AT skew value (–0.0168), indicating that T base was more common than A base, whereas GC skewness was negative (–0.2728).

All the PCGs used the initiation codon ATG. CO1, ATP8, ATP6, CO3, ND4L, ND5, ND6, and CytB used TAA terminal as the termination codon; ND1, ND2, and ND3 used TAG terminal as the termination codon, and three incomplete termination codons (T) were found in the other genes (CO2 and ND4). Among the 13 PCGs, the content of Asp was the lowest, accounting for only 1.47%, and the five amino acids (from high to low: Leu 15.07%, Ser 10.84%, Pro 9.48%, Thr 6.06%, and Phe 5.04%) were frequently used.

The lengths of 12S ribosomal RNA and 16S ribosomal RNA were 950 bp and 1690 bp, which were both located in the typical positions between tRNA-Phe and tRNA-Leu (UUA), being separated by tRNA-Val. These results were similar to the previous reports in *Gephyrocharax atracaudatus* and *Nematobrycon palmeri* (Huang et al. 2019; Wang et al. 2020). The overall A + T content was 55.52%, the AT and GC skew values for the rRNAs were −0.0168 and −0.2728, respectively. Additionally, the length of control region (D-loop) was 1194 bp, ranging from 15,685 bp to 16,878 bp. Here in *P. normani*, the control region had 372 nucleotides for A, and 380 nucleotides for T, both of which accounted for 62.98% of the whole D-loop.

The maximum-likelihood (ML) tree showed that *P. normani* was most closely related to *Xenotoca eiseni* among all the Cyprinodontidae species in the analysis. In Figure 1, Cyprinodontiformes were divided into three branches, *Poropanchax* + *Xenotoca* + *Cyprinodon* + *Orestias* was resolved as the sister group of a clade formed by *Poecilia* + *Xiphophorus* + *Poeciliopsis* in the combined data set. Other genera had a further evolutionary relationship than these two groups. Our results suggested that, the phylogenetic placement of *P. normani* was well supported within Cyprinodontidae. Phylogenetic analysis helps to clarify the phylogenetic classification of *P. normani*.

**Figure 1.**
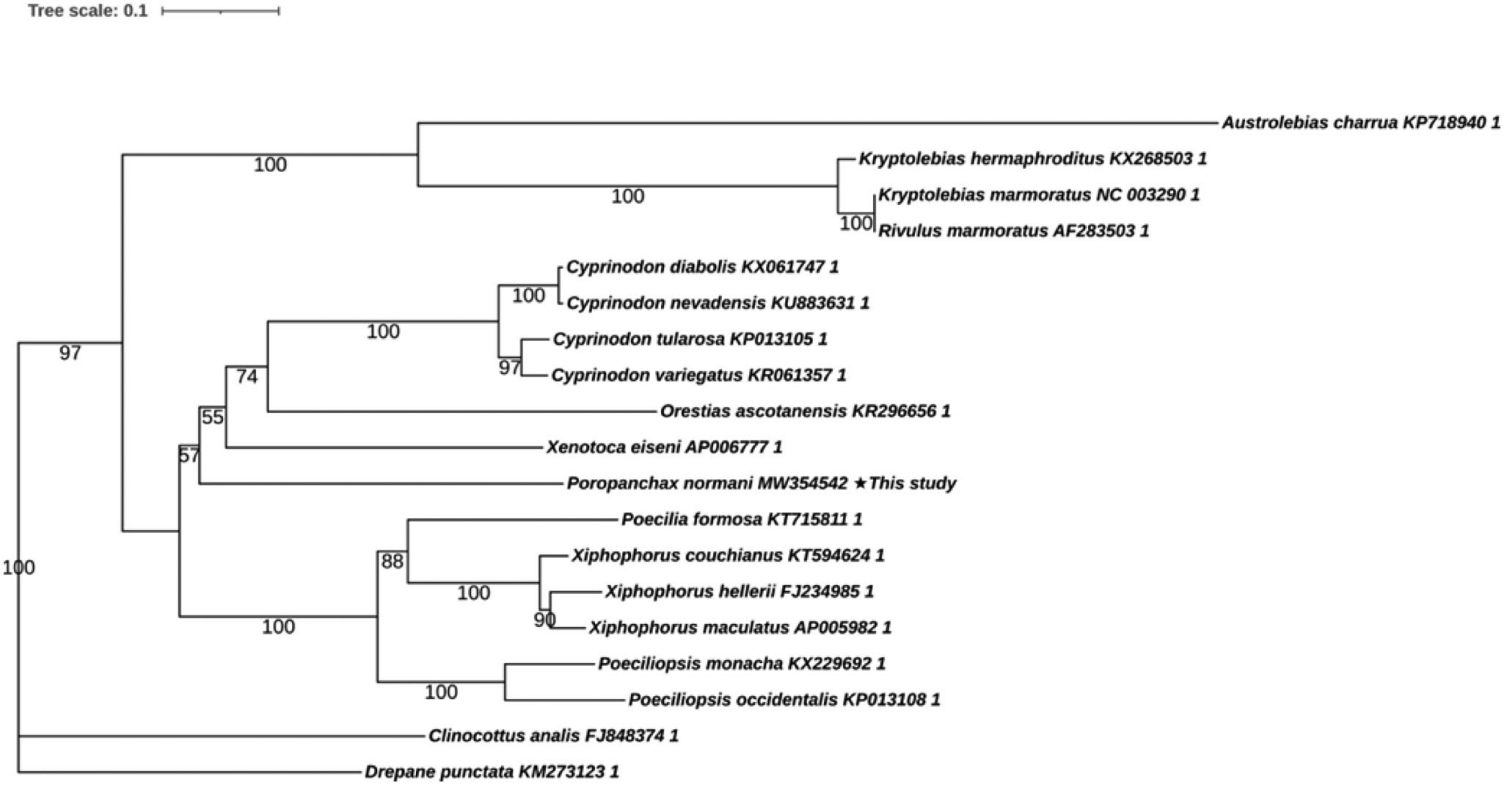
Maximum-likelihood (ML) tree of 17 Characidaes species and two outgroups based on 13 PCGs. The bootstrap values were based on 10,000 resamplings. The number at each node is the bootstrap probability. The number before the species name is the GenBank accession number.

We believe that our findings and more similar discoveries of these species would largely enhance the understanding of the Cyprinodontidae family.

## Supplementary Material

Supplemental Material

## Data Availability

The data that support the findings of this study are openly available at NCBI (https://www.ncbi.nlm.nih.gov), GenBank accession no. MW354542. The data that support the findings of this study are also available from the corresponding author, Dr. Yang, upon reasonable request.
