# Supplementary material for: The complete mitochondrial genome of Poropanchax normani and phylogenetic studies of Cyprinodontidaes
Source: Mitochondrial DNA B Resour. 2021 Aug 31;6(10):2796–8. doi: 10.1080/23802359.2021.1970633 (PMC8425730; doi:10.1080/23802359.2021.1970633)
Supplement: Supplemental Material [file TMDN_A_1970633_SM7633.doc]

Table S1. Sequences of the primers used in this study

| Primer name | Primer sequence (5′−3′) |
| --- | --- |
| PN-F0 | TTACACATGCAAGYCTCCGC |
| PN-R0 | CAGCGTTCCCTTGCGGTACTT |
| PN-F1 | GGTAAGTGTACCGGAAGGTG |
| PN-R1 | TCGTTGAACAAACGAACCCTT |
| PN-F2 | AAGACGAGAAGACCCTATGGAG |
| PN-R2 | GAADGGBCCYCCNGCRTAYTCTAC |
| PN-F3 | GCCGCACTTCTGTCAATA |
| PN-R3 | TGTAGCATAGTCGTAGGTAGA |
| PN-F4 | CCCTCACCCTACTATCACT |
| PN-R4 | AGAAGTAAGACGGCTGTG |
| PN-F5 | AGACCRAGGGCCTTCAAAG |
| PN-R5 | TARCTTCARTATCATTGRTGKCC |
| PN-F6 | GTAGTAGCMCACTTYCACTAYGT |
| PN-R6 | TGTGCTTGGTGGGCCATT |
| PN-F7 | AGTATTCACCGCCATTCTC |
| PN-R7 | GGACTAACCGCCTATTCG |
| PN-F8 | CTACAATGCTAAAARTYCTHATCCC |
| PN-R8 | TAGCCYHTGCTTGGATTTGCACCAAGAGT |
| PN-F9 | AACCACAACTGAGCCTCT |
| PN-R9 | TGTATTGATGATGCGGATGA |
| PN-F10 | CTACTGCCATTCACATCCA |
| PN-R10 | AAGCCATTAGTCTCGGTTAG |
| PN-F11 | CCGAGACTAATGGCTTGAA |
| PN-R11 | TCTGGCACTAGGAAGGATT |
| PN-F12 | ATGAATCGGAGGAATACCAGT |
| PN-R12 | CACTGCTGAATTCCCTTGGGGGTGTG |
